# Surgical Intervention in Very Elderly Patients with Spinal Ependymoma: A National Cancer Database Analysis

**DOI:** 10.3390/cancers18121927

**Published:** 2026-06-13

**Authors:** Garin Griffith, Saud K. Zaidan, Jacob Gould, Saarang Patel, Hazem S. Ghaith, Julian Gendreau, Maryam N. Shahin, Josiah N. Orina

**Affiliations:** 1 Department of Neurological Surgery, Oregon Health & Science University, Portland, OR 97239, USA; 2 Grossman School of Medicine, New York University, New York, NY 10016, USA; 3 Department of Biological Sciences, Seton Hall University, South Orange, NJ 07079, USA

**Keywords:** geriatric neurosurgery, National Cancer Database, overall survival, spinal cord neoplasms, spinal ependymoma, surgical resection

## Abstract

Background/Objectives: Spinal ependymoma is the most common intramedullary spinal cord tumor in adults, and maximal safe resection is the cornerstone of treatment. Patients aged 75 years and older are underrepresented in surgical neuro-oncology cohorts. We sought to characterize treatment patterns and identify predictors of overall survival in very elderly patients with spinal ependymoma. Methods: We performed a retrospective cohort study of patients aged 65 years or older with spinal ependymoma using the National Cancer Database. The primary cohort was patients aged 75 years or older (very elderly); patients aged 65–74 years served as a comparison cohort. Multivariable Cox proportional-hazards models were fit within each cohort, and a surgery-by-age-cohort interaction was tested. Results: Of 1497 eligible patients aged 65 years or older with spinal ependymoma, 422 patients (28.2%) met criteria for the final analytic cohort. Intramedullary versus extramedullary tumor status was not available in the NCDB PUF and therefore could not be characterized. Very elderly patients were less likely to undergo surgery than the comparison cohort (70% vs. 85%; *p* < 0.001) despite similar tumor characteristics. Among very elderly patients, median overall survival was 59.7 months without surgery and 106.0 months with surgery, an approximately 46-month difference favoring surgery. Surgery was independently associated with lower mortality (HR 0.46; 95% CI, 0.24–0.89; *p* = 0.021). Increasing age (HR 1.15 per year; 95% CI, 1.07–1.22; *p* < 0.001), Charlson–Deyo score ≥ 2 (HR 4.41; 95% CI, 1.65–11.79; *p* = 0.003), and increasing tumor size (HR 1.02 per mm; 95% CI, 1.01–1.04; *p* < 0.001) were also independently associated with worse survival. In the 65–74 cohort, no significant association between surgery and overall survival was detected (HR 1.23; 95% CI, 0.54–2.81; *p* = 0.623), though statistical power was limited by only 7 deaths in the no-surgery arm. The surgery-by-age-cohort interaction was significant (HR 0.37; *p* = 0.043). Conclusions: Surgical resection was independently associated with improved overall survival in very elderly patients with spinal ependymoma despite lower utilization. Chronological age alone may be an imperfect basis for excluding older adults from surgical consideration.

## Simple Summary

Spinal ependymoma is the most common tumor that grows inside the spinal cord in adults, and surgery to remove the tumor is the main treatment. However, older patients, especially those aged 75 years and older, are rarely studied separately, and it is unclear whether they benefit from surgery in the same way as younger patients. We reviewed records from a large national cancer registry to compare outcomes in patients aged 65 to 74 years with those aged 75 years and older. We found that patients aged 75 and older were less likely to be offered surgery, even though their tumors looked similar to those in younger patients. When they did undergo surgery, they lived substantially longer than those who did not. These findings suggest that age alone should not be the reason to avoid surgery, and that older patients may benefit more than is currently recognized.

## 1. Introduction

Ependymomas are glial neoplasms arising from ependymal cells lining the ventricular system and the central canal of the spinal cord. In adults, spinal ependymomas are the most common intramedullary tumor, accounting for more than 60% of intramedullary lesions [1,2]. The 2021 World Health Organization classification of CNS tumors organizes ependymal neoplasms by anatomic compartment and molecular features, recognizing molecularly defined supratentorial, posterior fossa, and spinal subtypes, while myxopapillary ependymoma is now designated CNS WHO grade 2 rather than grade 1 [3,4]. Adult spinal ependymomas are most often histologically low-grade and carry a relatively favorable prognosis when gross total resection is achieved [1,5].

Maximal safe resection is the cornerstone of treatment, and extent of resection is consistently identified as a primary determinant of progression-free and, in some cohorts, overall survival [5,6,7,8,9]. In a recent multicenter SEER analysis of 1580 adults with spinal ependymoma, 5- and 10-year survival rates were 96.7% and 95.4%, and age ≥65 years was an independent predictor of mortality (HR 3.93) [7]. The role of adjuvant radiotherapy in low-grade, gross totally resected tumors remains debated, with prior National Cancer Database (NCDB) data showing no overall survival benefit in grade II–III spinal ependymoma [10]. Most published outcome data, however, are derived from cohorts skewed toward younger and middle-aged adults, and their applicability to the very elderly is unclear.

The US population is aging, and the largest projected increase in cancer incidence between 2015 and 2050 is among adults aged ≥75 years [11]. Patients aged 75 and older are systematically underrepresented in surgical neuro-oncology cohorts, and treatment decisions in this group are frequently shaped by concerns about comorbidity, frailty, and limited functional reserve rather than tumor biology alone. In an NSQIP analysis of 4662 spinal tumor surgery patients, frailty was a more robust predictor of postoperative mortality (AUC 0.743) than chronological age (AUC 0.594), suggesting age alone is an imperfect proxy for surgical risk [12,13]. Whether the conservative approach often adopted in the very elderly is justified by comparable outcomes, or represents undertreatment, has not been adequately studied. Existing population-level studies generally pool all adult age groups or focus on the broader ≥65 category without separately characterizing patients aged 75 and older, and age-specific predictors of survival in the very elderly remain incompletely defined [14].

To address this gap, we conducted an NCDB analysis of patients aged 65 and older with spinal ependymoma, with the very elderly cohort aged 75 and older as the primary cohort of interest and a 65–74-year comparison cohort included to contextualize age-specific patterns. Our objectives were to characterize baseline clinical, demographic, and treatment patterns by age cohort, evaluate the association between surgical management and overall survival, and identify independent predictors of survival in the very elderly.

## 2. Materials and Methods

### 2.1. Data Source

A retrospective cohort study was performed using the National Cancer Database (NCDB; 2004–2022) central nervous system Participant User File. The NCDB is a hospital-based oncology registry jointly sponsored by the American College of Surgeons Commission on Cancer and the American Cancer Society that captures patients diagnosed and/or treated at Commission on Cancer-accredited facilities. Because the NCDB contains de-identified registry data, this study was considered exempt from institutional review board oversight and informed consent requirements.

### 2.2. Patient Selection

Patients with spinal ependymoma were identified using ICD-O-3 primary site and histology codes. Spinal primary sites were defined as C72.0 for spinal cord and C72.1 for cauda equina. Ependymoma histologies included 9391 for ependymoma, NOS; 9392 for anaplastic ependymoma; 9393 for papillary ependymoma; 9394 for myxopapillary ependymoma; and 9396 for molecularly defined ependymoma codes when available.

Patients were included if they were aged 65 years or older at diagnosis. The primary cohort of interest was the very elderly cohort, defined a priori as patients aged 75 years or older. Patients aged 65–74 years were included as a younger elderly comparison cohort to contextualize whether associations observed in the very elderly cohort were also present among younger older adults.

Patients were excluded from the primary analysis if they had missing or invalid survival time, missing vital status, missing or ambiguous surgical classification, missing tumor size, missing required model covariates, missing or unknown radiotherapy status, or survival time recorded as zero months. A cohort selection flow diagram was generated to describe sample attrition from the eligible age-restricted spinal ependymoma cohort to the final analytic cohort.

### 2.3. Surgical Classification

Surgical management was classified using the NCDB summary surgical procedure of the primary site variables. For diagnoses before 2023, RX_SUMM_SURG_PRIM_SITE was used; for diagnoses in 2023 and later, RX_SUMM_SURG_PRIM_SITE_2023 was used when available. A harmonized surgical variable was created to maximize capture across coding eras. The primary exposure was surgical management, with no surgery defined by codes 00 or A000 and surgery defined by codes 22 or A220, corresponding to resection of a tumor of the spinal cord or spinal nerve.

Codes corresponding to local excision or biopsy, ambiguous brain extent-of-resection codes appearing in spinal primary sites, tumor destruction, surgery not otherwise specified, unknown surgery, or missing surgery were excluded from the primary surgical analysis. Because NCDB spinal surgery coding does not reliably distinguish subtotal from gross total resection, extent of resection was not assessed as subtotal resection versus gross total resection.

### 2.4. Covariates

Demographic variables included age at diagnosis, sex, race, ethnicity, and insurance status. Clinical variables included Charlson–Deyo comorbidity score, primary site, histology, radiotherapy status, tumor size, and survival status. Facility and access variables included facility type, median household income quartile, education quartile, urban/rural residence, distance from patient residence to treating facility, and year of diagnosis.

Charlson–Deyo comorbidity score was modeled categorically as 0, 1, and 2 or more [15,16]. Year of diagnosis was retained as a continuous calendar-year covariate. Tumor size was harmonized across legacy and modern NCDB coding eras. For diagnoses through 2015, the legacy TUMOR_SIZE variable was used. For diagnoses in 2016 and later, the modern tumor size summary variables were prioritized, using TUMOR_SIZE_SUMMARY_16 when available and TUMOR_SIZE_SUMMARY_2016 as a fallback. Values were interpreted in millimeters; invalid, unknown, or registry-specific non-size codes were set to missing. The harmonized tumor size variable was modeled continuously per 1-mm increase.

For baseline descriptive tables, histology was presented using granular histologic categories. For multivariable modeling, histology was collapsed into myxopapillary ependymoma versus non-myxopapillary ependymoma to improve model stability. Race was collapsed as White versus non-White. Insurance was modeled as Medicare versus non-Medicare/other. Facility type was modeled as academic/research program versus non-academic/other. Residence was modeled as metro versus non-metro. Median household income was modeled as the highest quartile versus lower quartiles, and education was modeled as the highest quartile versus lower quartiles.

Radiotherapy was included as a treatment covariate only when it could be classified without missingness. Patients were classified as having received radiotherapy if radiation treatment was documented. Patients were classified as not having received radiotherapy only when no radiotherapy was explicitly coded. Patients with unknown or missing radiotherapy status were excluded from the primary complete-case–cohort. Tumor grade was evaluated but excluded from the primary analysis because of extensive missingness. Chemotherapy was excluded because it was rarely administered and provided minimal analytic variation.

### 2.5. Outcomes

The primary outcome was overall survival, defined as months from diagnosis to death from any cause or last follow-up. Patients alive at last follow-up were censored. Vital status was coded using PUF_VITAL_STATUS, with NCDB code 0 treated as death and code 1 treated as alive. Death was treated as the event. Patients with missing vital status, missing survival time, unknown elapsed survival time, or survival time recorded as zero months were excluded from the primary analysis.

### 2.6. Statistical Analysis

Baseline demographic, clinical, facility, treatment, socioeconomic, and outcome characteristics were summarized by age cohort. Continuous variables were reported as median [IQR], and categorical variables were reported as frequency with percentage. Percentages were rounded to the nearest whole number except for values below 5% or above 95%, which were reported with one decimal place. Group comparisons were performed using Wilcoxon rank-sum tests for continuous variables and chi-square or Fisher exact tests for categorical variables, as appropriate.

To address potential selection bias from complete-case–cohort construction, baseline characteristics were compared between patients included in the final analytic cohort and those excluded from the eligible age-restricted cohort. This comparison is presented in Table S1.

Kaplan–Meier curves were generated separately for the very elderly cohort and the younger elderly comparison cohort to compare overall survival between patients who underwent surgery and those who did not. Survival differences were assessed using the log-rank test, and number-at-risk tables were displayed beneath Kaplan–Meier curves.

All primary inferential analyses were centered on the very elderly cohort. A parallel analysis was performed in the younger elderly comparison cohort aged 65–74 years to contextualize age-specific patterns in treatment utilization and survival. Separate multivariable Cox proportional-hazards models were constructed for the very elderly cohort and the younger elderly comparison cohort. The primary model included surgical management, age, sex, race, ethnicity, insurance status, median household income, education, urban/rural residence, facility type, primary site, histology group, radiotherapy status, year of diagnosis, Charlson–Deyo comorbidity score, distance to facility, and tumor size. Tumor size was modeled per 1-mm increase. Hazard ratios with 95% confidence intervals were reported. The proportional hazards assumption was assessed using Schoenfeld residuals.

To evaluate the robustness of the primary findings to a less saturated model specification, parsimonious Cox models were fit within each age cohort, adjusting for surgical treatment, age, sex, Charlson–Deyo comorbidity score, tumor size, histology group, and radiotherapy status. As an exploratory analysis, a surgery-by-age-cohort interaction test was performed in the combined cohort to evaluate whether the association between surgery and overall survival differed between the very elderly cohort and the younger elderly comparison cohort. A likelihood-ratio test compared otherwise similar Cox models with and without the surgery-by-age-cohort interaction term. Analyses were performed in R version 4.5.2 using RStudio version 2026.1.0.392. Statistical significance was defined as a two-sided *p*-value less than 0.05.

## 3. Results

### 3.1. Patient Characteristics

**Figure 1:**
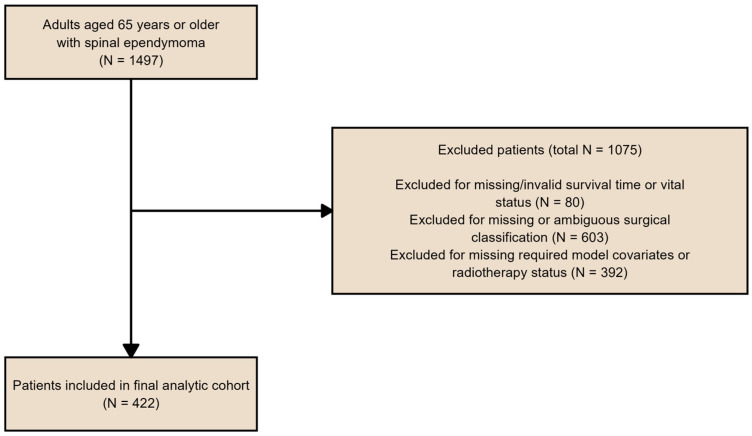
Patients aged 65 years or older with spinal ependymoma were identified in the National Cancer Database. Patients were sequentially excluded for missing or invalid survival time or vital status, missing or ambiguous surgical classification, and missing required model covariates or radiotherapy status. The final analytic cohort comprised 422 patients, including 126 in the very elderly cohort (≥75 years) and 296 in the younger elderly comparison cohort (65–74 years).

Among 1497 patients aged 65 years or older with spinal ependymoma, 1075 were excluded during cohort construction, including 80 patients with missing or invalid survival time or vital status, 603 with missing or ambiguous surgical classification, and 392 with missing required model covariates or radiotherapy status. The final analytic cohort included 422 patients (Figure 1).

Baseline characteristics of included and excluded patients are shown in Table S1. Included and excluded patients were similar with respect to age, age cohort, sex, facility type, primary site, histology, Charlson–Deyo comorbidity score, and distance to facility. However, included patients were more commonly diagnosed in later calendar years (median 2016 vs. 2013; *p* < 0.001), had lower median tumor size (19.5 mm vs. 22.0 mm; *p* = 0.015), and differed with respect to ethnicity, insurance, income, education, residence, surgical treatment classification, and radiotherapy status. Differences in surgical treatment classification and tumor size missingness are expected given that missing or ambiguous surgery and missing tumor size were exclusion criteria.

The primary very elderly cohort included 126 patients aged 75 years or older, while 296 patients aged 65–74 years comprised the younger elderly comparison cohort (Table 1). Median age was 78 years [IQR, 76–82 years] in the very elderly cohort and 68 years [IQR, 66–71 years] in the comparison cohort (*p* < 0.001). Sex distribution differed numerically but did not reach statistical significance, with a higher proportion of female patients in the very elderly cohort compared with the comparison cohort (58% vs. 48%; *p* = 0.077).

Demographic, socioeconomic, facility-level, and tumor characteristics were broadly similar between the very elderly cohort and the younger elderly comparison cohort. Most patients were White in both the very elderly and comparison cohorts (94% vs. 93%; *p* > 0.900), and most were non-Hispanic (95.2% vs. 98.0%; *p* = 0.196). Insurance status, median household income quartile, education quartile, residence, facility type, primary tumor site, and histology did not differ significantly between cohorts. The majority of tumors arose in the spinal cord rather than the cauda equina in both the very elderly and comparison cohorts (97.6% vs. 95.9%; *p* = 0.568). Ependymoma, NOS was the most common histology, followed by myxopapillary ependymoma.

Treatment patterns differed between the very elderly cohort and the younger elderly comparison cohort. Very elderly patients were less likely to undergo surgical resection than patients in the comparison cohort (70% vs. 85%), while no surgery was more common in the very elderly cohort (30% vs. 15%; *p* < 0.001). Radiotherapy use did not differ significantly between cohorts and was documented in 13% of very elderly patients and 17% of patients in the comparison cohort (*p* = 0.348). Median year of diagnosis, Charlson–Deyo comorbidity score distribution, distance to treating facility, and tumor size were similar between cohorts. Median tumor size was 20.5 mm [IQR, 12.0–36.0 mm] in the very elderly cohort and 19.0 mm [IQR, 12.0–29.5 mm] in the comparison cohort (*p* = 0.169). Death events were more common in the very elderly cohort than in the younger elderly comparison cohort (45% vs. 20%; *p* < 0.001).

### 3.2. Kaplan–Meier Survival Analysis

**Figure 2:**
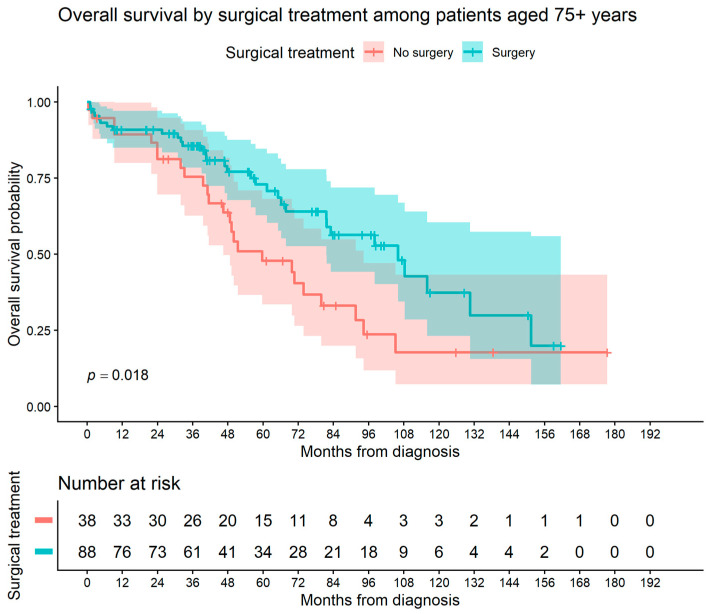
Median overall survival was 106.0 months among patients who underwent surgical resection (*n* = 88) and 59.7 months among those managed without surgery (*n* = 38), with separation of survival curves over follow-up (log-rank *p* = 0.018). The number-at-risk table is displayed beneath the survival curves.

**Figure 3:**
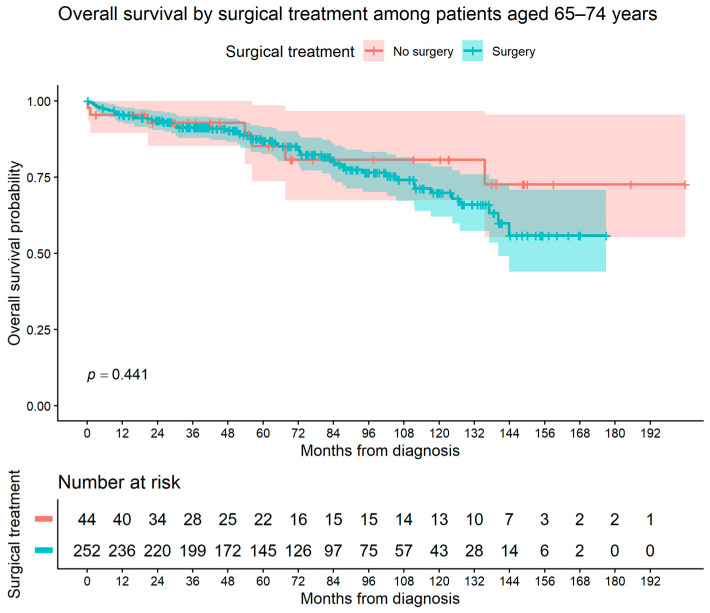
Survival trajectories overlapped between patients who underwent surgical resection (*n* = 252) and those managed without surgery (*n* = 44), and no statistically significant difference in overall survival was observed (log-rank *p* = 0.441). The number-at-risk table is displayed beneath the survival curves.

In the primary very elderly cohort, Kaplan–Meier analysis demonstrated a significant difference in overall survival by surgical treatment status (Figure 2). Median overall survival was 106.0 months among patients who underwent surgery and 59.7 months among those managed without surgery, corresponding to an approximately 46-month median survival difference favoring surgery (log-rank *p* = 0.018). The 95% CI for median survival was 81.6 months to not reached in the surgery group and 46.4–94.3 months in the no-surgery group. In the younger elderly comparison cohort, median overall survival was not reached in either the surgery or no-surgery group, and no statistically significant difference in overall survival by surgical treatment status was observed (log-rank *p* = 0.441; Figure 3).

### 3.3. Multivariable Cox Regression Analysis

In the primary multivariable Cox model for the very elderly cohort, surgical resection was associated with lower mortality compared with no surgery (Table 2). After adjustment for demographic, socioeconomic, facility-level, tumor, and treatment covariates, surgery was associated with a lower hazard of death (HR 0.46; 95% CI, 0.24–0.89; *p* = 0.021).

Increasing age was independently associated with worse overall survival in the very elderly cohort, with each additional year of age associated with higher mortality risk (HR 1.15; 95% CI, 1.07–1.22; *p* < 0.001). Charlson–Deyo comorbidity score of 2 or greater was also associated with higher mortality compared with a score of 0 (HR 4.41; 95% CI, 1.65–11.79; *p* = 0.003). Tumor size was independently associated with mortality, with each 1-mm increase in tumor size associated with a higher hazard of death (HR 1.02; 95% CI, 1.01–1.04; *p* < 0.001). Myxopapillary histology demonstrated a trend toward lower mortality compared with non-myxopapillary ependymoma, though this did not meet statistical significance (HR 0.54; 95% CI, 0.28–1.02; *p* = 0.057). Sex, race, ethnicity, insurance status, income, education, residence, facility type, primary site, radiotherapy, year of diagnosis, and distance to facility were not independently associated with overall survival in the very elderly cohort.

In contrast, surgery was not associated with overall survival in the younger elderly comparison cohort (HR 1.23; 95% CI, 0.54–2.81; *p* = 0.623) (Table 2). Increasing age remained associated with worse survival in this cohort (HR 1.12 per year; 95% CI, 1.02–1.22; *p* = 0.018). Charlson–Deyo comorbidity score of 2 or greater was also associated with increased mortality compared with a score of 0 (HR 2.23; 95% CI, 1.07–4.61; *p* = 0.031). Tumor size was independently associated with overall survival, although the effect estimate was small when modeled per 1-mm increase (HR 1.003; 95% CI, 1.001–1.005; *p* = 0.006). No other covariates, including sex, race, ethnicity, insurance status, income, education, residence, facility type, primary site, histology, radiotherapy, year of diagnosis, or distance to facility, were significantly associated with survival in the younger elderly comparison cohort.

No global proportional hazards violations were identified in either age-stratified model, and no proportional hazards violation was observed for the primary surgical exposure.

### 3.4. Parsimonious Model and Interaction Analysis

In parsimonious sensitivity models adjusting for surgical treatment, age, sex, Charlson–Deyo comorbidity score, tumor size, histology group, and radiotherapy status, the primary findings were directionally consistent (Table S2). In the very elderly cohort, surgery remained associated with lower mortality compared with no surgery (HR 0.44; 95% CI, 0.24–0.79; *p* = 0.006). In the younger elderly comparison cohort, surgery was not associated with overall survival (HR 1.26; 95% CI, 0.57–2.79; *p* = 0.575).

In an exploratory interaction analysis, the surgery-by-age-cohort interaction term was statistically significant (HR 0.37; 95% CI, 0.14–0.97; *p* = 0.043), and a likelihood-ratio test comparing models with versus without the interaction term was also significant (χ^2^ = 4.49; *p* = 0.034) (Table S3). These exploratory findings suggest that the association between surgery and overall survival differed between the very elderly cohort and the younger elderly comparison cohort.

## 4. Discussion

In this NCDB analysis of 422 patients aged 65 years and older with spinal ependymoma, we found that surgical resection was independently associated with lower overall mortality among very elderly patients aged 75 years and older but was not associated with overall survival among the younger elderly comparison cohort aged 65 to 74 years. The age-specific divergence in the surgical association was supported by an exploratory surgery-by-age-cohort interaction test, in which the interaction term and a likelihood-ratio test comparing nested models were both significant. In addition to surgery, increasing age, Charlson–Deyo comorbidity score of 2 or greater, and increasing tumor size were associated with worse overall survival in the very elderly cohort. Notably, very elderly patients were substantially less likely to undergo surgical resection than patients in the comparison cohort despite similar tumor characteristics, raising the possibility that age alone may be driving treatment decisions in a population that could still derive a measurable survival benefit from surgery.

Our findings are consistent with the prior literature establishing surgical resection as a primary determinant of survival in adult spinal ependymoma, while extending those findings into a population that is rarely studied separately [5,6,7,14]. Single-institution series and earlier population-based analyses have generally reported favorable long-term outcomes after gross total resection in younger and middle-aged adults, with the extent of resection emerging as a stronger prognostic factor than histologic grade in many cohorts [5,6,7,8,17,18]. In a comprehensive review of 175 adult spinal ependymoma patients with clearly defined WHO grade, grade I and II tumors did not differ significantly in outcomes following surgery, while gross total resection provided significantly improved progression-free and overall survival compared with subtotal resection [8]. Our median overall survival of 106.0 months in surgically managed very elderly patients is consistent with prior surgical series; Lin et al. reported a median survival of 104 months following complete resection alone in adult spinal cord ependymoma [19]. Most of these reports either pool all adult age groups or exclude older adults entirely, and few separately characterize patients aged 75 years and older.

The absence of a survival association with surgery in the 65–74-year cohort likely reflects several factors rather than a true lack of benefit. Low-grade adult spinal ependymoma is relatively indolent, and only 20% of this cohort died during follow-up, limiting statistical power. The high baseline surgical rate (85%) constrained the no-surgery comparator to a small group (*n* = 44, 7 deaths) that likely included patients with unmeasured contraindications or frailty, potentially biasing the comparison toward the null. Younger elderly patients also have greater physiologic reserve and longer competing-cause life expectancy, which may dilute a short- to intermediate-term tumor-specific survival benefit. The null finding should not be interpreted as evidence against surgical resection in this group, but as a reflection of overall survival being an insensitive endpoint in a predominantly surgical, low-event population.

The observation that very elderly patients were nearly twice as likely as their younger elderly counterparts to forgo surgical resection (30% vs. 15%) is clinically important. Demographic, socioeconomic, facility-level, and tumor characteristics, including primary site, histology, tumor size, comorbidity score distribution, and facility type, were broadly similar between cohorts, suggesting that age itself, rather than measured differences in disease biology or access to care, is a principal driver of nonoperative management. The combination of lower surgical utilization with a stronger surgery-associated survival benefit in the very elderly raises the concern that some older patients with spinal ependymoma may be undertreated. This concern is reinforced by NSQIP data showing that frailty, not chronological age, is the dominant predictor of postoperative mortality in spinal tumor surgery, implying that risk-stratified surgical offerings rather than age-based exclusion may better serve this population [12]. At the same time, our analysis cannot fully account for unmeasured determinants of surgical candidacy, including baseline neurologic function, performance status, frailty, and patient or family preferences, all of which legitimately influence the decision to operate on an older adult with an intramedullary spinal tumor.

Within the very elderly cohort, comorbidity burden and tumor size emerged as additional independent predictors of overall survival. A Charlson–Deyo comorbidity score of 2 or greater was associated with more than a fourfold increase in the hazard of death compared with a score of 0, and each 1-mm increase in tumor size was associated with a small but significant incremental increase in mortality risk. Each additional year of age was also independently associated with worse survival within the 75-and-older cohort, indicating that prognostic heterogeneity persists even within the very elderly subgroup. Together, these findings suggest that comorbidity status, tumor size, and age may help identify very elderly patients in whom surgical resection is most likely to improve survival, and conversely, those for whom expected gains may be more limited. Myxopapillary histology demonstrated a trend toward lower mortality compared with non-myxopapillary ependymoma in the very elderly cohort, although this association did not reach statistical significance and should be interpreted as hypothesis-generating; this directional signal is consistent with the generally favorable long-term prognosis reported in dedicated SEER analyses of spinal myxopapillary ependymoma [20].

The lack of an independent association between radiotherapy and overall survival in either age cohort is consistent with the broader uncertainty surrounding adjuvant radiation in adult spinal ependymoma, particularly for low-grade disease [10,21,22]. A prior NCDB analysis of 1058 adults with WHO grade II–III spinal ependymoma similarly found that adjuvant radiotherapy did not reduce the hazard of death for the cohort overall or among grade II tumors [10]. Radiotherapy was uncommon in both of our cohorts (13% in the very elderly and 17% in the comparison group), and our analysis was not designed to evaluate radiotherapy effects within specific extent-of-resection or histologic subgroups. Similarly, demographic, socioeconomic, and facility-level variables, including race, ethnicity, insurance status, household income quartile, education quartile, residence, facility type, and distance to facility, were not significantly associated with overall survival within either age cohort. While reassuring on the surface, these null findings should be interpreted in the context of a relatively homogeneous and predominantly insured older adult population and limited statistical power for subgroup comparisons.

From a clinical standpoint, our findings support a more individualized approach to surgical decision-making in older adults with spinal ependymoma. Rather than using chronological age alone as a barrier to surgery, treatment recommendations may be better informed by an integrated assessment of comorbidity burden, tumor size, baseline neurologic function, and patient goals of care [12,23,24]. Frailty assessment using validated, multidimensional instruments has been advocated for perioperative risk stratification in older neurosurgical patients, and is increasingly viewed as complementary to chronological age [23]. The combination of a strong adjusted survival association with surgery in the very elderly, a significant surgery-by-age-cohort interaction, and consistency between primary and parsimonious models suggests that the observed benefit is unlikely to be explained entirely by model specification or residual confounding from a small set of measured covariates. Multidisciplinary evaluation that incorporates frailty assessment, geriatric oncology input, and a careful discussion of perioperative risks and expected functional outcomes may help align surgical offerings with the subset of very elderly patients most likely to benefit.

Beyond overall survival, quality of life and functional outcomes are central considerations in surgical decision-making for patients aged 75 and older. Intramedullary spinal cord surgery in this population carries risks including new motor or sensory deficits, urinary and bowel dysfunction, prolonged rehabilitation, and loss of independent ambulation; even temporary postoperative decline can precipitate institutionalization [25]. Conversely, untreated spinal ependymoma can itself produce devastating neurologic morbidity, and the survival benefit observed in our very elderly surgical cohort must be weighed against the realistic trajectory of nonoperative management. Future studies pairing survival with neurologic function, ambulatory status, and patient-reported quality of life are needed to fully inform shared decision-making in this population [26].

### Limitations

Several limitations should be considered when interpreting our findings. First, this is a retrospective observational analysis of registry data, and the association between surgery and overall survival is susceptible to residual and unmeasured confounding. Patients selected for surgery may have differed systematically from those managed nonoperatively in ways that the NCDB does not capture, including baseline neurologic function, ambulatory status, performance status, frailty, anesthetic risk, cognitive function, tumor location within the spinal cord, extent of cord involvement, and patient or family preferences. Selection bias in surgical candidacy is a particular concern in older adults [27]. Patients selected for resection in our very elderly cohort were almost certainly healthier and more functional than those managed nonoperatively along these unmeasured dimensions, inflating the magnitude of the observed surgery-associated hazard ratio. The HR of 0.46 should therefore be interpreted as an upper bound on the potential survival benefit rather than a causal effect estimate.

Second, the NCDB does not reliably distinguish gross total from subtotal resection for spinal tumors, and we therefore assessed surgery as a binary exposure rather than by extent of resection. Because extent of resection is a well-established prognostic factor in adult spinal ependymoma, our binary classification likely underestimates the true heterogeneity of surgical outcomes [5,6,8]. The registry also lacks granular detail on tumor location along the neuraxis, intramedullary versus extramedullary status, neurologic deficit at presentation, recurrence, progression, and disease-specific mortality, all of which limit the granularity of our outcome assessment. Overall survival was the only available outcome, and progression-free survival, neurologic outcomes, and quality of life could not be evaluated.

Third, missing data necessitated a complete-case analytic strategy. From an eligible cohort of 1497 patients aged 65 years or older, 1075 were excluded for missing or invalid survival data, missing or ambiguous surgical classification, or missing model covariates, leaving a final analytic cohort of 422. We therefore compared baseline characteristics of included and excluded patients in Table S1. Although included and excluded patients were similar across several core demographic and tumor variables, they differed by year of diagnosis, tumor size, ethnicity, insurance, socioeconomic measures, residence, surgical treatment classification, and radiotherapy status. These differences suggest that selection bias may remain despite complete-case analytic clarity, particularly because missingness was not random across registry eras and key treatment variables [28]. Tumor grade was excluded from the primary analysis owing to extensive missingness, and chemotherapy was not analyzable because it was rarely administered.

Fourth, the very elderly cohort included 126 patients with 57 death events, which limits statistical power for fully adjusted multivariable modeling and constrains the precision of our hazard ratio estimates. Wide confidence intervals around several covariates and the borderline significance of myxopapillary histology illustrate this limitation. The surgery-by-age-cohort interaction analysis was prespecified as exploratory and should be regarded as hypothesis-generating rather than confirmatory. Additionally, although the NCDB captures approximately 73.7% of newly diagnosed cancers in the United States, it is restricted to Commission on Cancer-accredited facilities, which may limit the generalizability of our findings to community and non-accredited care settings [29,30].

Finally, our analytic cohort was predominantly non-Hispanic White (approximately 94% White and 97% non-Hispanic), which is more skewed than reported in some neuropathological or single-institution clinical series of spinal ependymoma. Several factors likely contribute to this pattern. The U.S. population aged 65 years and older is substantially more non-Hispanic White than the general population, reflecting historical demographic trends, and our age-restricted cohort inherits this baseline imbalance. Complete-case attrition for missing covariates may have also preferentially retained patients treated at facilities with more complete data capture, which can vary by patient demographics [29]. Additionally, CBTRUS data indicate that incidence of spinal ependymoma is highest among non-Hispanic White individuals, so the cohort distribution partly reflects underlying disease epidemiology rather than purely selection artifact [17]. Nonetheless, the limited representation of Black, Hispanic, Asian/Pacific Islander, and American Indian/Alaska Native patients constrains the generalizability of our findings across racial and ethnic groups and limits our ability to detect race- or ethnicity-specific patterns in treatment utilization or survival. Future studies that intentionally oversample underrepresented populations are needed to address this gap.

## 5. Conclusions

Very elderly patients with spinal ependymoma were substantially less likely to undergo surgery than patients aged 65–74 years, despite similar measured tumor characteristics. In this cohort, surgical resection was independently associated with improved overall survival, with an approximately 46-month median survival difference favoring surgery. These findings raise concern for possible age-based undertreatment, while emphasizing that operative decisions in patients aged 75 years or older should balance expected survival benefit against quality of life, frailty, neurologic function, comorbidity burden, and patient goals.

## Figures and Tables

**Table 1:** Baseline demographic, socioeconomic, clinical, and treatment characteristics of patients aged 65 years or older with spinal ependymoma, stratified by age cohort.

Characteristic	Overall ^1^ *n* = 422	75+ ^1^ *n* = 126	65–74 ^1^ *n* = 296	*p*-Value ^2^
Age, years	71 [67, 76]	78 [76, 82]	68 [66, 71]	<0.001
Sex				0.077
Male	207 (49%)	53 (42%)	154 (52%)	
Female	215 (51%)	73 (58%)	142 (48%)	
Race				>0.9
White	395 (94%)	119 (94%)	276 (93%)	
Black	19 (4.5%)	5 (4.0%)	14 (4.7%)	
Asian/Pacific Islander	6 (1.4%)	2 (1.6%)	4 (1.4%)	
American Indian/Alaska Native	1 (0.2%)	0 (0.0%)	1 (0.3%)	
Other race *	1 (0.2%)	0 (0.0%)	1 (0.3%)	
Ethnicity				0.196
Not Hispanic	410 (97.2%)	120 (95.2%)	290 (98.0%)	
Hispanic	12 (2.8%)	6 (4.8%)	6 (2.0%)	
Insurance				0.207
Medicare	358 (85%)	113 (90%)	245 (83%)	
Private insurance	48 (11%)	10 (8%)	38 (13%)	
Medicaid	5 (1.2%)	0 (0.0%)	5 (1.7%)	
Other government **	7 (1.7%)	1 (0.8%)	6 (2.0%)	
Not insured	4 (0.9%)	2 (1.6%)	2 (0.7%)	
Median Household Income Quartile				0.786
Q4 highest income	165 (39%)	46 (37%)	119 (40%)	
Q3	108 (26%)	36 (29%)	72 (24%)	
Q2	95 (23%)	29 (23%)	66 (22%)	
Q1 lowest income	54 (13%)	15 (12%)	39 (13%)	
Education Quartile				0.102
Q4 highest education	116 (28%)	42 (33%)	74 (25%)	
Q3	155 (37%)	36 (29%)	119 (40%)	
Q2	95 (23%)	32 (25%)	63 (21%)	
Q1 lowest education	56 (13%)	16 (13%)	40 (14%)	
Residence				0.361
Metro	344 (82%)	100 (79%)	244 (82%)	
Urban	70 (17%)	25 (20%)	45 (15%)	
Rural	8 (1.9%)	1 (0.8%)	7 (2.4%)	
Facility Type				0.149
Academic/Research Program	198 (47%)	50 (40%)	148 (50%)	
Comprehensive Community Cancer Program	127 (30%)	42 (33%)	85 (29%)	
Integrated Network Cancer Program	87 (21%)	29 (23%)	58 (20%)	
Community Cancer Program	10 (2.4%)	5 (4.0%)	5 (1.7%)	
Primary Site				0.568
Spinal cord	407 (96.4%)	123 (97.6%)	284 (95.9%)	
Cauda equina	15 (4%)	3 (2.4%)	12 (4.1%)	
Histology				0.222
Ependymoma, NOS	239 (57%)	76 (60%)	163 (55%)	
Myxopapillary ependymoma	175 (42%)	46 (37%)	129 (44%)	
Papillary ependymoma	5 (1.2%)	2 (1.6%)	3 (1.0%)	
Anaplastic ependymoma	3 (0.7%)	2 (1.6%)	1 (0.3%)	
Surgical Resection				<0.001
No surgery	82 (19%)	38 (30%)	44 (15%)	
Surgery	340 (81%)	88 (70%)	252 (85%)	
Radiotherapy				0.348
Not performed	356 (84%)	110 (87%)	246 (83%)	
Performed	66 (16%)	16 (13%)	50 (17%)	
Year of diagnosis	2016 [2013, 2019]	2016 [2013, 2019]	2016 [2013, 2020]	0.458
Charlson–Deyo comorbidity score				0.433
0	299 (71%)	94 (75%)	205 (69%)	
1	83 (20%)	20 (16%)	63 (21%)	
2	28 (7%)	7 (6%)	21 (7%)	
3+	12 (2.8%)	5 (4.0%)	7 (2.4%)	
Distance to facility, miles	17.2 [6.7, 40.0]	14.3 [5.7, 34.9]	17.6 [7.2, 41.1]	0.131
Tumor size, mm	19.5 [12.0, 30.0]	20.5 [12.0, 36.0]	19.0 [12.0, 29.5]	0.169
Death events				<0.001
Overall	117 (28%)	57 (45%)	60 (20%)	

^1^ Continuous variables presented as median [Q1, Q3]; categorical variables presented as *n* (%). ^2^ Wilcoxon rank-sum test for continuous variables; Pearson chi-square or Fisher exact test for categorical variables. * Other race includes American Indian/Aleutian/Eskimo, Hawaiian, Micronesian NOS, Pacific Islander NOS, and Other race NOS. ** Other government insurance includes TRICARE, Military, Veterans Affairs, and Indian/Public Health Service.

**Table 2:** Multivariable Cox proportional-hazards models of overall survival, stratified by age cohort, among patients with spinal ependymoma aged 75 years or older (*n* = 126) and 65–74 years (*n* = 296).

	75+ Years (*n* = 126)		65–74 Years (*n* = 296)	
Characteristic	HR (95% CI)	*p*-Value	HR (95% CI)	*p*-Value
Age (per 1 year)	1.15 (1.07–1.22)	**<0.001**	1.12 (1.02–1.22)	**0.018**
Sex				
Male	-		-	
Female	0.96 (0.51–1.79)	0.891	0.76 (0.44–1.32)	0.33
Race				
White	-		-	
Non-White	0.62 (0.16–2.34)	0.481	0.26 (0.04–1.75)	0.166
Ethnicity				
Not Hispanic	-		-	
Hispanic	1.58 (0.44–5.70)	0.482	1.87 (0.41–8.46)	0.418
Insurance				
Medicare	-		-	
Non-Medicare/other	1.09 (0.41–2.93)	0.859	0.68 (0.28–1.65)	0.397
Median Household Income				
Q4 highest income	-		-	
Q1–Q3 lower income quartiles	0.73 (0.34–1.53)	0.402	1.42 (0.69–2.94)	0.344
Education				
Q4 highest education	-		-	
Q1–Q3 lower education quartiles	1.06 (0.56–2.00)	0.867	0.87 (0.42–1.83)	0.717
Residence				
Metro	-		-	
Non-metro	1.29 (0.57–2.91)	0.536	1.21 (0.60–2.45)	0.602
Facility Type				
Academic/Research Program	-		-	
Non-academic/other	1.43 (0.74–2.78)	0.291	1.18 (0.68–2.05)	0.567
Primary Site				
Spinal cord	-		-	
Cauda equina	0.94 (0.18–4.83)	>0.9	1.43 (0.42–4.90)	0.566
Histology				
Non-myxopapillary ependymoma	-		-	
Myxopapillary ependymoma	0.54 (0.28–1.02)	0.057	0.76 (0.43–1.33)	0.332
Surgical Resection				
No surgery	-		-	
Surgery	0.46 (0.24–0.89)	**0.021**	1.23 (0.54–2.81)	0.623
Radiotherapy				
Not performed	-		-	
Performed	0.66 (0.27–1.63)	0.368	1.14 (0.58–2.23)	0.713
Year of diagnosis (per 1 year)	1.04 (0.94–1.13)	0.459	1.05 (0.96–1.15)	0.325
Charlson–Deyo score				
0	-		-	
1	0.63 (0.27–1.48)	0.293	0.76 (0.36–1.59)	0.463
2+	4.41 (1.65–11.79)	**0.003**	2.23 (1.07–4.61)	**0.031**
Distance to facility (per 10 miles)	0.99 (0.95–1.03)	0.574	0.98 (0.92–1.05)	0.537
Tumor size (per 1 mm)	1.02 (1.01–1.04)	**<0.001**	1.003 (1.001–1.005)	**0.006**

HR, hazard ratio; CI, confidence interval. Models adjusted for surgical management, age, sex, race, ethnicity, insurance status, median household income, education, urban/rural residence, facility type, primary site, histology group, radiotherapy, year of diagnosis, Charlson–Deyo comorbidity score, distance to facility, and tumor size. Tumor size was modeled per 1-mm increase. Reference categories are indicated by a hyphen(-). Bolded *p*-values denote statistical significance at *p* < 0.05.

## Data Availability

The data used in this study were obtained from the National Cancer Database Participant User File. The NCDB Participant User File contains HIPAA-compliant, de-identified patient-level data and is available through application to the American College of Surgeons National Cancer Database for investigators associated with Commission on Cancer-accredited cancer programs. Data are not publicly available from the authors because use of the NCDB Participant User File is governed by a data use agreement.

## Supplementary Materials

The following supporting information can be downloaded at: https://www.mdpi.com/article/10.3390/cancers18121927/s1, Table S1: Baseline Characteristics of Included and Excluded Patients in the Age-Restricted Spinal Ependymoma Cohort; Table S2: Parsimonious Cox proportional-hazards models of overall survival, stratified by age cohort; Table S3: Interaction analysis for differential association between surgery and overall survival by age cohort.

## Abbreviations

The following abbreviations are used in this manuscript:

AUC	Area under the curve
CI	Confidence interval
CNS	Central nervous system
HR	Hazard ratio
ICD-O-3	International Classification of Diseases for Oncology, 3rd Edition
IQR	Interquartile range
NCDB	National Cancer Database
NOS	Not otherwise specified
NSQIP	National Surgical Quality Improvement Program
SEER	Surveillance, Epidemiology, and End Results
WHO	World Health Organization
