# Supplementary material for: Surgical Intervention in Very Elderly Patients with Spinal Ependymoma: A National Cancer Database Analysis
Source: Cancers (Basel). 2026 Jun 13;18(12):1927. doi: 10.3390/cancers18121927 (PMC13297340; doi:10.3390/cancers18121927)
Supplement: Supplementary file 1 [file cancers-18-01927-s001.zip › cancers-4355645-supplementary.pdf]

Supplemental Table S1. Baseline Characteristics of Included and Excluded Patients in the Age-Restricted Spinal Ependymoma Cohort

Characteristic	Overall N = 1,497[1]	Included N = 422[1]	Excluded N = 1075[1]	p-value[2]
**Age, years**	70 [67, 75]	71 [67, 76]	70 [67, 75]	0.881
Missing/unknown Age, years	0 (0%)	0 (0%)	0 (0%)	
**Age cohort**				0.243
75+	413 (28%)	126 (30%)	287 (27%)	
65-74	1084 (72%)	296 (70%)	788 (73%)	
**Sex**				>0.9
Male	734 (49%)	207 (49%)	527 (49%)	
Female	763 (51%)	215 (51%)	548 (51%)	
**Race**				0.090
White	1373 (92%)	395 (94%)	978 (91%)	
Black	67 (4.5%)	19 (4.5%)	48 (4.5%)	
Asian/Pacific Islander	27 (1.8%)	6 (1.4%)	21 (2.0%)	
American Indian/Alaska Native	4 (0.3%)	1 (0.2%)	3 (0.3%)	
Other race	13 (0.9%)	1 (0.2%)	12 (1.1%)	
Missing/unknown	13 (0.9%)	0 (0%)	13 (1.2%)	
**Ethnicity**				**<0.001**
Not Hispanic	1371 (92%)	410 (97.2%)	961 (89%)	
Hispanic	73 (4.9%)	12 (2.8%)	61 (6%)	
Missing/unknown	53 (3.5%)	0 (0%)	53 (4.9%)	
**Insurance**				**0.013**
Medicare	1242 (83%)	358 (85%)	884 (82%)	
Private insurance	189 (13%)	48 (11%)	141 (13%)	
Medicaid	22 (1.5%)	5 (1.2%)	17 (1.6%)	
Other government*	17 (1.1%)	7 (1.7%)	10 (0.9%)	
Not insured	8 (0.5%)	4 (0.9%)	4 (0.4%)	
Missing/unknown	19 (1.3%)	0 (0%)	19 (1.8%)	
**Median household income quartile**				**<0.001**
Q4 highest income	543 (36%)	165 (39%)	378 (35%)	
Q3	328 (22%)	108 (26%)	220 (20%)	
Q2	255 (17%)	95 (23%)	160 (15%)	
Q1 lowest income	175 (12%)	54 (13%)	121 (11%)	
Missing/unknown	196 (13%)	0 (0%)	196 (18%)	
**Education quartile**				**<0.001**
Q4 highest education	422 (28%)	116 (27%)	306 (28%)	
Q3	381 (25%)	155 (37%)	226 (21%)	
Q2	307 (21%)	95 (23%)	212 (20%)	

Q1 lowest education	191 (13%)	56 (13%)	135 (13%)	
Missing/unknown	196 (13%)	0 (0%)	196 (18%)	
**Residence**				**<0.001**
Metro	1169 (78%)	344 (82%)	825 (77%)	
Urban	236 (16%)	70 (17%)	166 (15%)	
Rural	24 (1.6%)	8 (1.9%)	16 (1.5%)	
Missing/unknown	68 (4.5%)	0 (0%)	68 (6%)	
**Facility type**				0.692
Academic/Research Program	671 (45%)	198 (47%)	473 (44%)	
Comprehensive Community Cancer Program	482 (32%)	127 (30%)	355 (33%)	
Integrated Network Cancer Program	306 (20%)	87 (21%)	219 (20%)	
Community Cancer Program	38 (2.5%)	10 (2.4%)	28 (2.6%)	
**Primary site**				0.622
Spinal cord	1436 (95.9%)	407 (96.4%)	1029 (95.7%)	
Cauda equina	61 (4.1%)	15 (3.6%)	46 (4.3%)	
**Histology**				0.784
Ependymoma, NOS	867 (58%)	239 (57%)	628 (58%)	
Myxopapillary ependymoma	602 (40%)	175 (41%)	427 (40%)	
Papillary ependymoma	14 (0.9%)	5 (1.2%)	9 (0.8%)	
Anaplastic ependymoma	14 (0.9%)	3 (0.7%)	11 (1.0%)	
**Surgical treatment**				**<0.001**
No surgery	178 (12%)	82 (19%)	96 (9%)	
Surgery	706 (47%)	340 (81%)	366 (34%)	
Missing/unknown	613 (41%)	0 (0%)	613 (57%)	
**Radiotherapy**				**0.037**
Not performed	1222 (82%)	356 (84%)	866 (81%)	
Performed	264 (18%)	66 (16%)	198 (18%)	
Missing/unknown	11 (0.7%)	0 (0%)	11 (1.0%)	
**Year of diagnosis**	2015 [2010, 2019]	2016 [2013, 2019]	2013 [2008, 2018]	**<0.001**
Missing/unknown, year of diagnosis	0 (0%)	0 (0%)	0 (0%)	
**Charlson-Deyo comorbidity score**				0.145
0	1037 (69%)	299 (71%)	738 (69%)	
1	279 (19%)	83 (20%)	196 (18%)	
2+	181 (12%)	40 (9%)	141 (13%)	
**Distance to facility, miles**	15.9 [6.7, 41.5]	17.2 [6.7, 40.0]	15.2 [6.8, 43.7]	>0.9
Missing/unknown, distance to facility, miles	195 (13%)	0 (0%)	195 (18%)	
**Tumor size, mm**	20.0 [13.0, 33.5]	19.5 [12.0, 30.0]	22.0 [14.0, 35.0]	**0.015**
Missing/unknown Tumor size, mm	545 (36%)	0 (0%)	545 (51%)	

[1]*Continuous variables presented as median [Q1, Q3]; categorical variables presented as n (%).*

[2]Wilcoxon rank-sum test for continuous variables; Pearson chi-square or Fisher exact test for categorical variables.

** Other government insurance includes TRICARE, Military, Veterans Affairs, and Indian/Public Health Service.*

Supplemental Table S2. Parsimonious Cox proportional-hazards models of overall survival, stratified by age cohort.

	75+ years (N = 126)		65-74 years (N = 296)	
**Characteristic**	**HR (95% CI)**	**p-value**	**HR (95% CI)**	**p-value**
**Age (per 1 year)**	1.14 (1.07-1.21)	**<0.001**	1.11 (1.02-1.21)	**0.021**
**Surgical treatment**				
No surgery	-		-	
Surgery	0.44 (0.24-0.79)	**0.006**	1.26 (0.57-2.79)	0.575
**Sex**				
Male	-		-	
Female	0.81 (0.46-1.42)	0.458	0.75 (0.44-1.28)	0.288
**Charlson-Deyo score**				
0	-		-	
1	0.63 (0.29-1.40)	0.256	0.75 (0.36-1.56)	0.442
2+	4.13 (1.69-10.07)	**0.002**	2.26 (1.14-4.48)	**0.019**
**Tumor size (per 1 mm)**	1.02 (1.01-1.03)	**<0.001**	1.02 (1.00-1.00)	**0.021**
**Histology**				
Non-myxopapillary ependymoma	-		-	
Myxopapillary ependymoma	0.56 (0.30-1.05)	0.069	0.76 (0.44-1.30)	0.315
**Radiotherapy**				
Not performed	-		-	
Performed	0.80 (0.36-1.79)	0.590	1.13 (0.58-2.18)	0.725

HR, hazard ratio; CI, confidence interval. Parsimonious models adjusted for surgical treatment, age, sex, Charlson-Deyo comorbidity score, tumor size, histology group, and radiotherapy status. Tumor size modeled per 1-mm increase. Bold values indicate statistical significance at p<0.05.

Supplemental Table S3. Interaction analysis for differential association between surgery and overall survival by age cohort.

Analysis	Result	p-value
Surgery × age-group interaction term	HR 0.37 (95% CI, 0.14-0.97)	**0.043**
Likelihood-ratio test comparing models with vs without surgery × age-group interaction	$\chi^2 = 4.49$	**0.034**

The interaction HR ratio compares the surgery-associated hazard ratio in the 75+ cohort with the surgery-associated hazard ratio in the 65–74 cohort. Values below 1.00 indicate a stronger inverse association between surgery and mortality in the 75+ cohort. The likelihood-ratio test compares otherwise similar Cox proportional-hazards models with and without the surgery-by-age-cohort interaction term.
